# Supplementary material for: Embryos and embryonic stem cells from the white rhinoceros
Source: Nat Commun. 2018 Jul 4;9:2589. doi: 10.1038/s41467-018-04959-2 (PMC6031672; doi:10.1038/s41467-018-04959-2)
Supplement: Supplementary file 1 — Supplementary Information [file 41467_2018_4959_MOESM1_ESM.pdf]

Supplementary Information

Embryos and embryonic stem cells from the white rhinoceros

(Hildebrandt et al)

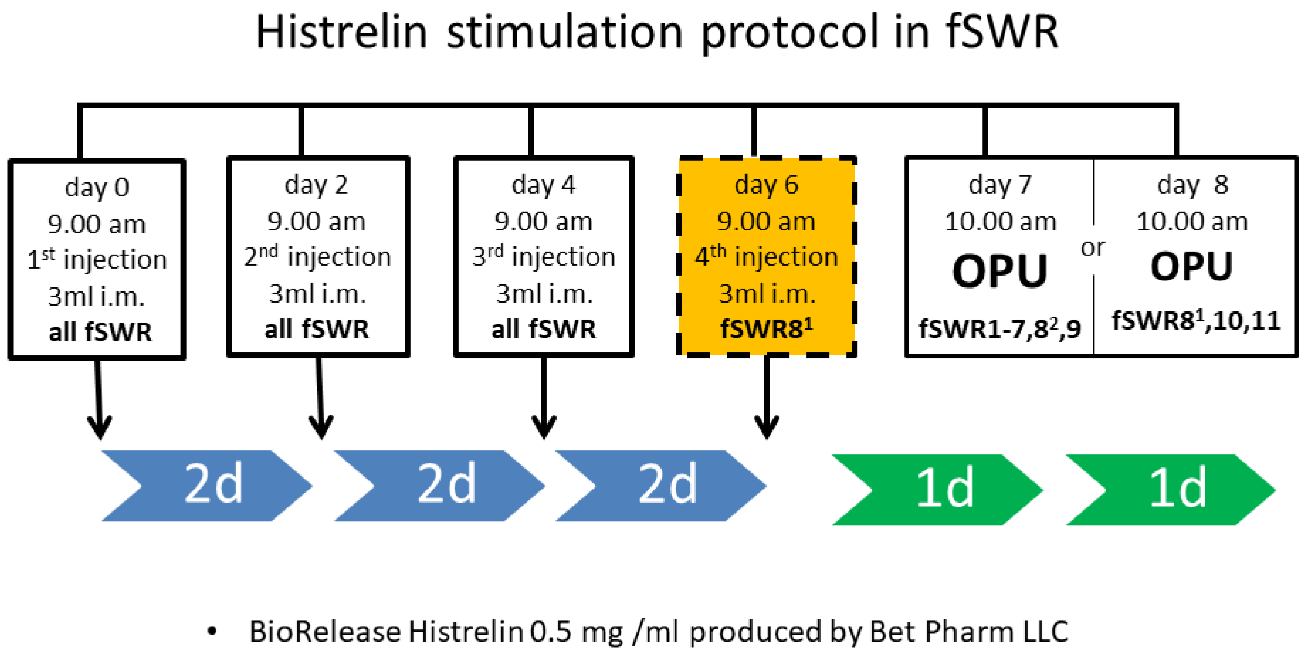

Supplementary Figure 1. GnRH stimulation protocol in fSWR

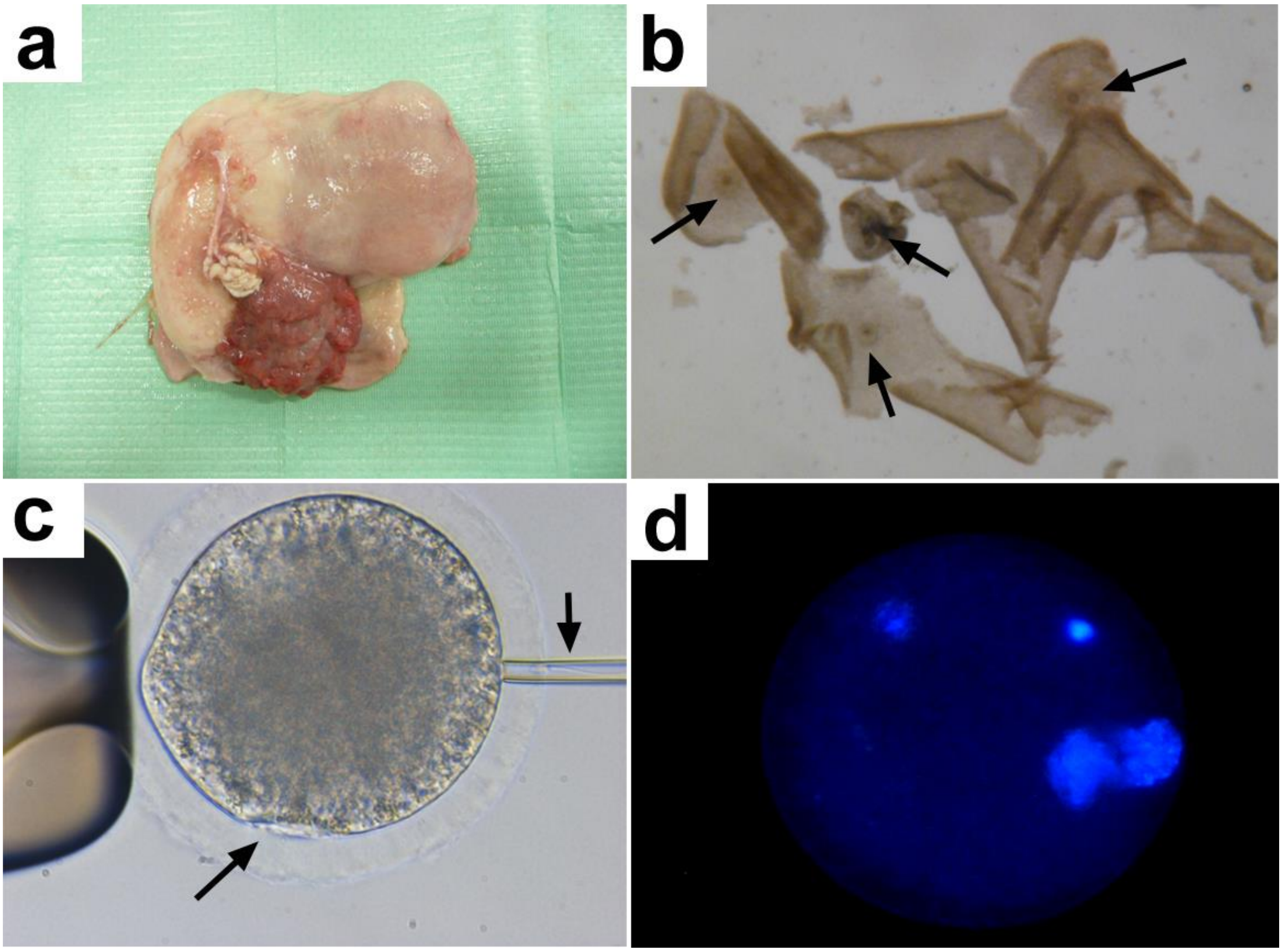

Supplementary Figure 2. Oocytes collected from a natural deceased NWR aged female (32 years old). One of the 2 ovaries pictured in **a**), very fibrotic with no follicles visible on the surface. **b**) after dissection and slicing of the ovaries few follicles were identified and 4 oocytes recovered, (arrows). **c**) one oocyte at the time of ICSI: the first poplar body is visible at 6 o'clock (arrow) and the ICSI pipette with the sperm inside is at 3 o'clock (arrow). **d**) fixed oocyte 72 h after ICSI that did not cleave, 2 pronuclei and two polar bodies were visible after Hoechst staining and fluorescence microscopy.

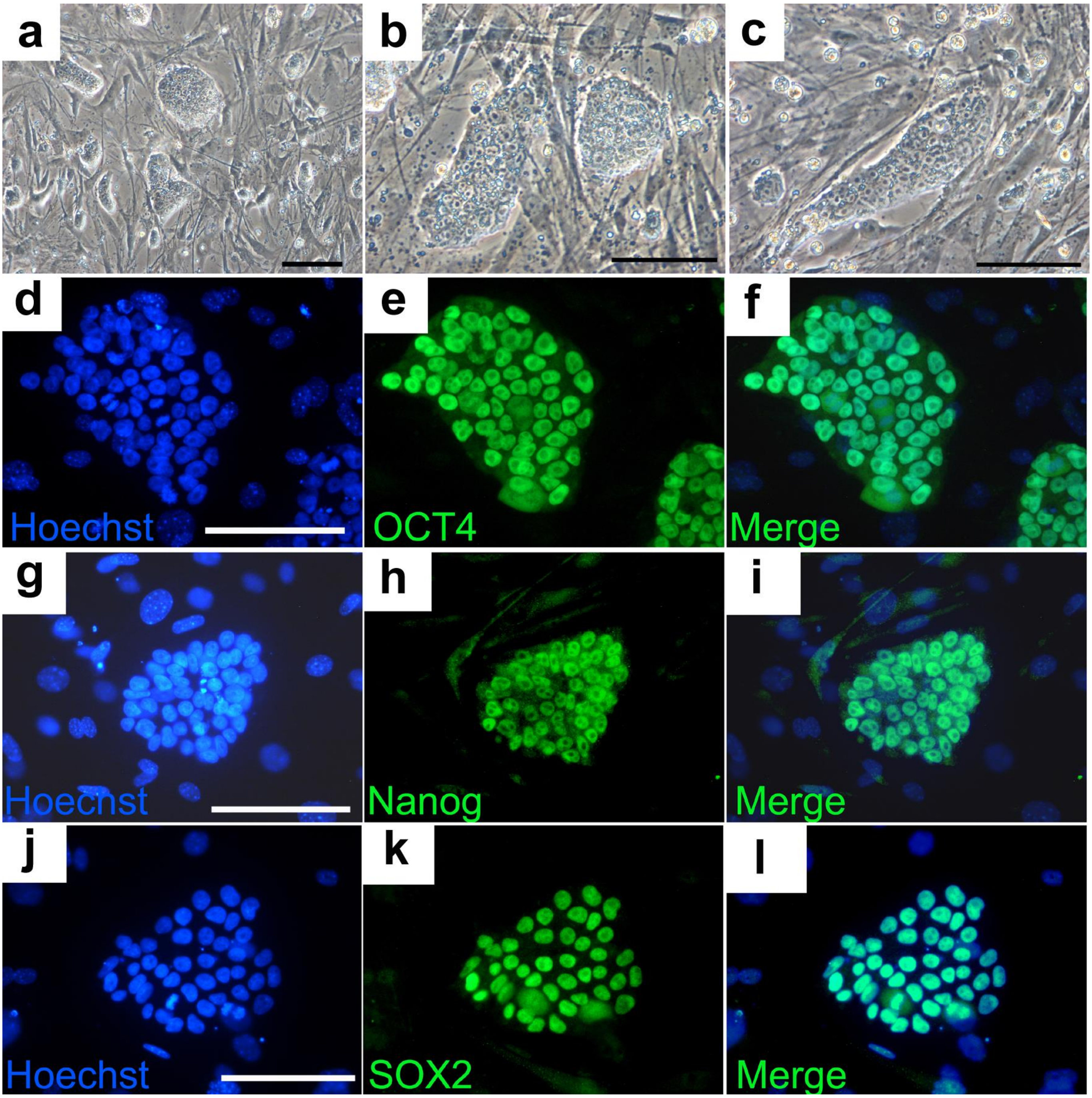

Supplementary Figure 3. rESC phase contrast and immunocytochemistry for pluripotency markers at higher magnification (40x). a) 20x and b, c) at 40x , phase contrast of growing colonies. d, e, f) staining for *OCT4*. g, h, i) staining for *NANOG*. j, k, l) staining for *SOX2*.

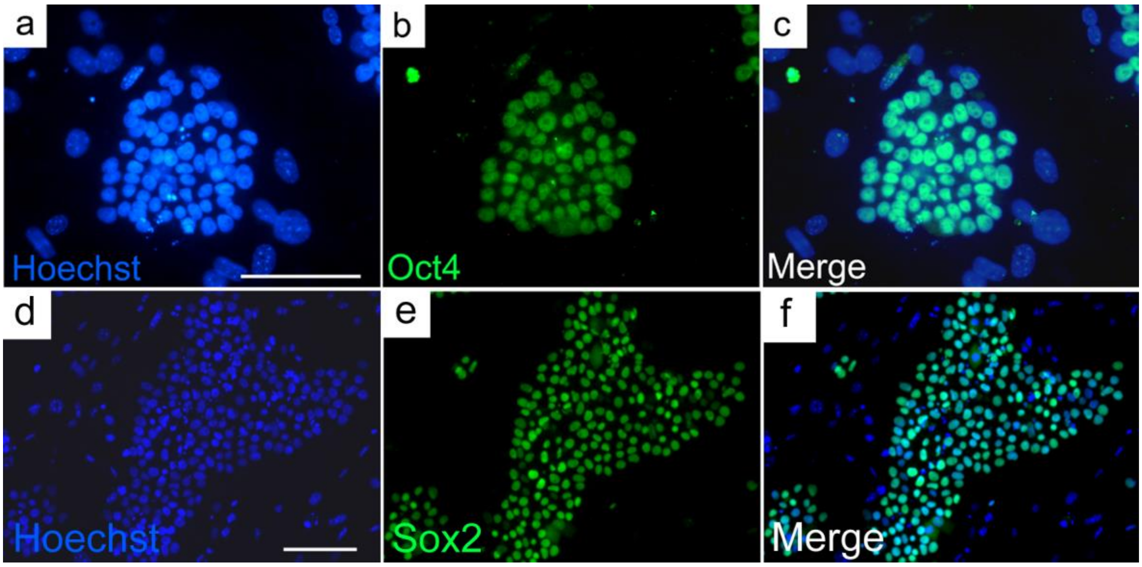

Supplementary Figure 4: Staining of rESC with monoclonal antibodies for pluripotency markers. **a**, **b**, **c**) *OCT4* (Mouse monoclonal, Santa Cruz, Oct3/4 sc-5279). **d**, **e**, **f**) *SOX2* (Mouse monoclonal, Thermofisher, MA-1-014)

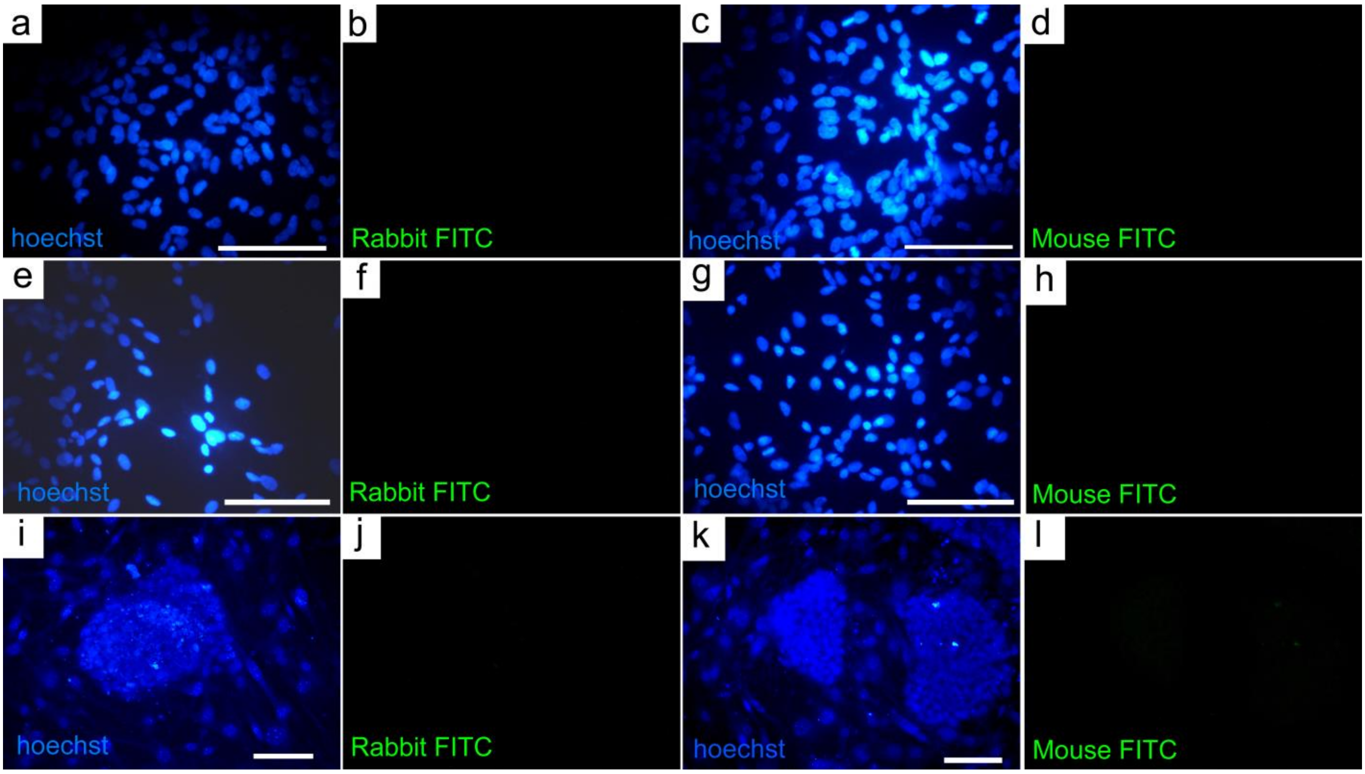

Supplementary Figure 5. Negative controls of secondary antibodies.
Following the same immunocytochemistry protocol, the primary antibodies (rabbit and mouse) were omitted. **a**,**b**, **c**, **d**) neural precursors; **e**, **f**, **g**, **h**) differentiated Neurons; **i**, **j**, **k**, **l**) undifferentiated rESC. It is clearly visible that no staining can be detected in the absence of the primary antibodies. Bar 100µm.

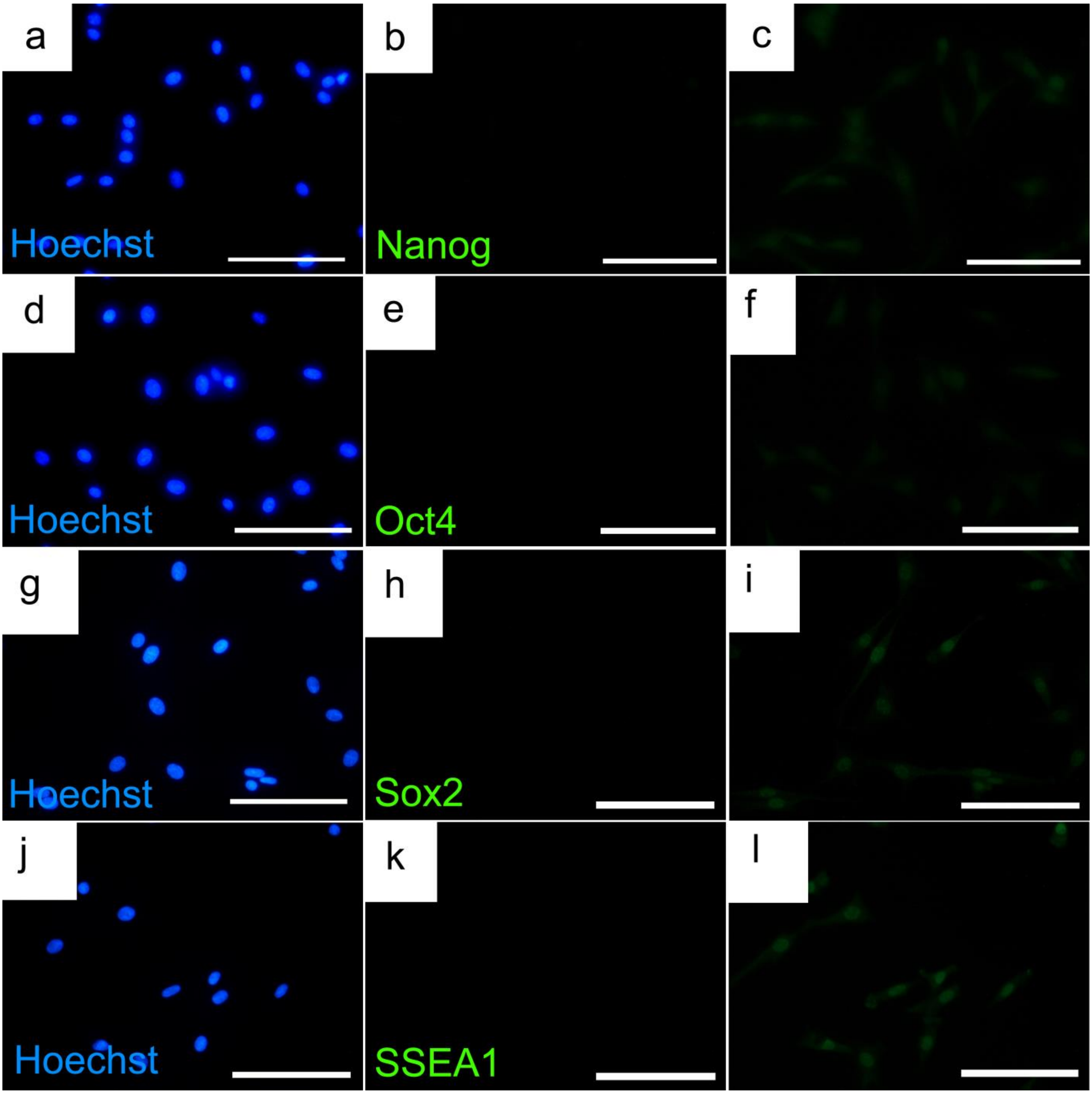

Supplementary Figure 6. Fibroblast specificity controls.
Rhino fibroblasts at passage 3 were plated on coverslips and then subjected to the same immunocytochemistry protocol used for the 4 undifferentiated markers to phenotype the two established rESC lines. **a**, **d**, **g**, **j)** Hoechst staining 0.5 seconds exposure; **b**, **e**, **h**, **k**) primary antibodies+ secondary antibodies 0.5 seconds exposure; **c**, **f**, **i**, **l**) 2 seconds exposure. It is clearly visible that the antibodies are not binding to fibroblasts. There is some weak staining in the overexposed picture (**l**) but SSEA1 should stain the surface of the cells and not the nucleus, so it is clearly background unspecific stain. Bar 100µm.

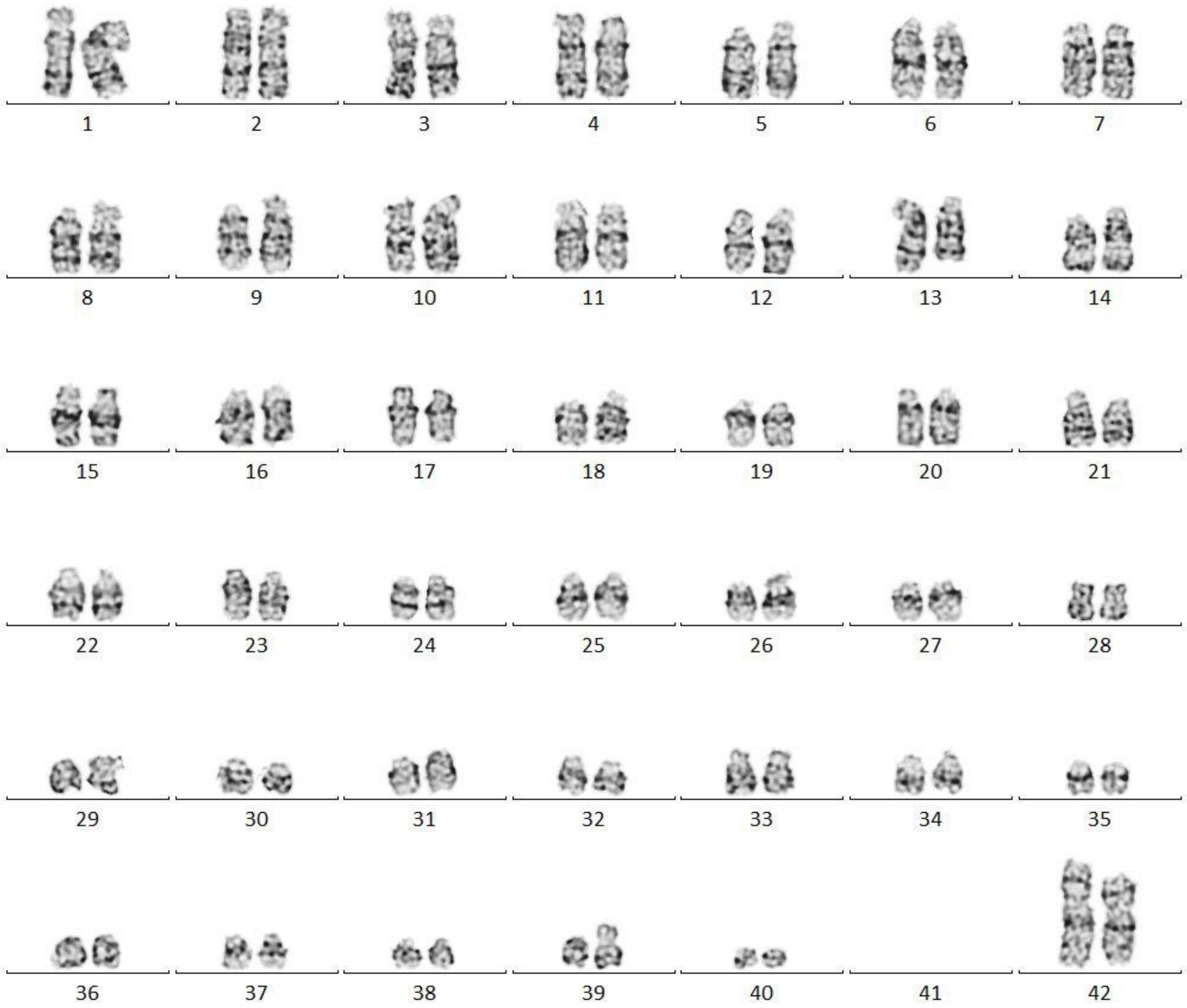

Supplementary Figure 7. Karyotype analysis of rESC. The analysis reveals the diploid female (sex Chr n 42 XX) status of the cell line analysed**.**

Supplementary Table 1. Pig ICSI test of semen sample of NWR and SWR bulls

Name	Breed	Motility Post Thaw (%)	Injected	Fixed/corr. fert. Cleaved	%
mNWR1	NWR	<10	24	9	38
			24	13	54
mNWR1*	NWR	<10	10	7	70
			10	9	90
mNWR2	NWR	<10	20	6	30
			27	10	37
mNWR2*	NWR	<10	10	9	90
			9	6	67
mNWR3	NWR	0	10	1	10
			10	1	10
mNWR3*	NWR	0	20	5	25
			10	3	30
mSWR1	SWR	40	20	17	85
			20	16	80
mSWR2	SWR	<10	20	12	60
			20	11	55
mSWR3	SWR	20	23	19	83
			25	5	20
mSWR4	SWR	<10	20	16	80
			20	15	75
mSWR5	SWR	40	20	18	90
			20	16	80
AOA** only	No Sperm	N.A.	20	1	5
			20	9***	45

* artificially activated

** artificial oocyte activation

*** in this case embryos were cultured for 7 days but all arrested at cleavage stages

Supplementary Table 2. **Primers used for the RT-PCR**

Gene	Forward	Reverse
SOX2	AACCCCAAGATGCACAACTC	CGGGGCCGGTATTTATAATC
NANOG	TCCAGCAGATGCAAGAACTTT	GCAAGTCTTTGGCCAGTTGT
OCT4	GCAGCAGATCAGCCACATC	CTCCCGTTGCGAATAGTCAT
18SRNA	GTAACCCGTTGAACCCCATT	CCATCCAATCGGTAGTAGCG

Supplementary Table 3: Oligonucleotides for paternity testing

No	Oligo Name	Scale	Method	Quality control	Purification	5'-Mod.	Type	Sequence (5'-3')
1	7B-F	40	High Purity	Normal no extra	HPLC	NONE	DNA	CCT CTG TGA TTA AGC AAG GC
2	7B-R	40	High Purity	Normal no extra	HPLC	HEX	DNA	ATG AAC AGG AAG GAA GAC GC
3	7C-F	40	High Purity	Normal no extra	HPLC	NONE	DNA	TGA ACT CTG ATG GAA ATG AG
4	7C-R	40	High Purity	Normal no extra	HPLC	HEX	DNA	AAC AGG TCT TGA TTA GTG C
5	DB1-F	40	High Purity	Normal no extra	HPLC	NONE	DNA	AGA TAA TAA TAG GAC CCT GCT CCC
6	DB1-R	40	High Purity	Normal no extra	HPLC	HEX	DNA	GAG GGT TTA TTG TGA ATG AGG C
7	DB44-F	40	High Purity	Normal no extra	HPLC	NONE	DNA	GGT GGA ATG TCA AGT AGC GG
8	DB44-R	40	High Purity	Normal no extra	HPLC	HEX	DNA	CTT GTT GCC CCA TCC CTG
9	DB49-F	40	High Purity	Normal no extra	HPLC	NONE	DNA	GTC AGG CAT TGG CAG GAA G
10	BR6-F	40	High Purity	Normal no extra	HPLC	NONE	DNA	TCA TTT CTT TGT TCC CCA TAG CAC
11	Rh7-F	40	High Purity	Normal no extra	HPLC	NONE	DNA	CCG TCA CAT ATG ACA GTG TGC
12	Rh9-F	40	High Purity	Normal no extra	HPLC	NONE	DNA	TCT GGT ACC ACC AAA TGT AGC
13	WR1-F	40	High Purity	Normal no extra	HPLC	NONE	DNA	GGC AAA ACT AAG AGA ACT TG
14	WR2-F	40	High Purity	Normal no extra	HPLC	NONE	DNA	ACA GCT AGA ATC ACC AAA AC
15	DB49-R	40	High Purity	Normal no extra	HPLC	HEX	DNA	CAG GGT AAG TGG GGG TGC
16	BR6-R	40	High Purity	Normal no extra	HPLC	HEX	DNA	AGC AAT ATC CAC GAT ATG TGA AGG
17	Rh7-R	40	High Purity	Normal no extra	HPLC	HEX	DNA	GGG CAG CTT ATG CTC AAG TC
18	Rh9-R	40	High Purity	Normal no extra	HPLC	FAM	DNA	ACG ATT ACG TCT TTC AGT TGC
19	WR1-R	40	High Purity	Normal no extra	HPLC	FAM	DNA	GAT ACC AAA CTG GAA ATG G
20	WR2-R	40	High Purity	Normal no extra	HPLC	FAM	DNA	TCC TGC TGC ATA AAT CTC

Supplementary Table 4: Oligonucleotides for sex determination

No	Oligo Name	Scale	Method	Quality control	Purification	5'-Mod.	Type	Sequence (5'-3')
1	ARZF1_f	40	High Purity	Normal no extra	HPLC	FAM	DNA	GAT TTG GAA SCT AGG CAT TTC C
2	ARZF1_r	40	High Purify	Normal no extra	HPLC	NONE	DNA	GCC ATG ATA CTC ATG AAT GAC A

Supplementary Table 5. Microsatellite analysis to confirm the parental origin and the sex of the embryos produced. 051A, 051B 240A and 240B are the cell derived from the corresponding 4 embryos

marker	SWR		SWR		SWR		x SWR		NWR		SWR		NWR		x SWR	
	mSWR1		fSWR5		051A		051B		mNWR2		fSWR5		240A		240B	
	1	2	1	2	1	2	1	2	1	2	1	2	1	2	1	2
7B	260	260	260	260	260	260	260	260	260	260	260	260	260	260	260	260
7C	251	253	251	251	251	253	251	253	249	251	251	251	249	251	249	251
BR6	133	154	133	154	133	154	133	154	126	126	133	154	126	133	126	154
DB1	130	130	130	130	130	130	130	130	123	127	130	130	127	130	123	130
DB44	167	175	169	169	167	169	169	175	179	181	169	169	169	179	169	181
DB49	158	161	158	161	161	161	158	161	163	163	158	161	161	163	158	163
RH7	193	193	193	193	193	193	193	193	193	193	193	193	193	193	193	193
RH9	136	138	136	138	136	136	138	138	136	148	136	138	136	136	138	148
WR1	178	188	178	188	178	178	178	188	182	188	178	188	182	188	188	188
WR2	225	225	225	239	225	239	225	239	235	235	225	239	225	235	235	239
ARZF1	96	103	96	96	96	96	96	96	96	103	96	96	96	96	96	103
	m		f		f		f		m		f		f		m	
