# Supplementary material for: Embryos and embryonic stem cells from the white rhinoceros
Source: Nat Commun. 2018 Jul 4;9:2589. doi: 10.1038/s41467-018-04959-2 (PMC6031672; doi:10.1038/s41467-018-04959-2)
Supplement: Supplementary file 3 — Description of Additional Supplementary Files [file 41467_2018_4959_MOESM3_ESM.pdf]

Description of Additional Supplementary Files

File Name: Supplementary Movie 1
Description: Beating cardiomyocytes obtained from direct differentiation in vitro of rES cell
